# Enhanced Blood Cell Detection in YOLOv11n Using Gradient Accumulation and Loss Reweighting

**DOI:** 10.3390/bioengineering12111188

**Published:** 2025-10-31

**Authors:** Min Feng, Juncai Xu

**Affiliations:** 1 The Affiliated Brain Hospital, Nanjing Medical University, Nanjing 210029, China; 2 School of Chinese Language and Literature, Nanjing Normal University, Nanjing 210024, China; 3 School of Engineering, Case Western Reserve University, Cleveland, OH 44106, USA; 4 College of Water Conservancy and Hydropower Engineering, Hohai University, Nanjing 210098, China

**Keywords:** blood cell detection, YOLO, gradient accumulation, loss reweighting

## Abstract

Automated blood cell detection is of significant importance in the efficient and accurate diagnosis of hematological diseases. The application of this technology has advanced clinical practice in hematology and improved the speed and accuracy of diagnosis, thereby providing patients with more timely medical intervention. In this study, the YOLOv11n model was optimized by integrating gradient accumulation and loss reweighting techniques to improve its detection performance for blood cells in clinical images. The optimized YOLOv11n model shows an improvement in performance. The mAP50 reached 0.9356, the mAP50-95 was 0.6620, and the precision and recall were better than those of existing methods. The model can effectively address issues such as dense cell distribution, cell overlap, and image artifacts. Therefore, it is highly applicable in real-time clinical applications. The results of the ablation experiment demonstrate that there is a synergistic effect between gradient accumulation and loss reweighting, which can improve detection accuracy without increasing the computational burden. The conclusion indicates that the optimized YOLOv11n model has important application prospects as an automated blood cell detection tool and has the potential to integrate with clinical workflows.

## 1. Introduction

Accurate blood cell detection is essential for a range of clinical applications, particularly in diagnosing hematological diseases such as anemia, leukemia, and infections [1]. Blood cells, including red blood cells (RBCs), white blood cells (WBCs), and platelets, are important biomarkers for assessing patients’ health and identifying disease [2]. Traditional manual microscopic blood smear analysis is time-consuming and error-prone, requiring specialized expertise [3]. Automated blood cell detection systems are expected to significantly improve diagnostic efficiency, consistency, and accuracy, thereby enhancing clinical decision-making and patient prognosis [4].

In recent years, advances in deep learning techniques have driven significant improvements in medical image analysis. Convolutional neural networks (CNNs) are highly effective in tasks such as organ segmentation, lesion detection, and disease classification, even in the presence of noise, poor image quality, and differences in acquisition conditions, which are common in clinical imaging [5,6]. In particular, the single-stage object detection architecture “You Only Look Once” (YOLO) has attracted considerable attention in the field of medical image analysis due to its efficiency in real-time, high-precision detection [7]. YOLO was first proposed by Redmon et al. and was recently reviewed. YOLOv1 pioneered a unified one-shot detection framework that achieves real-time operation, and subsequent versions have maintained real-time performance [7,8]. In the field of medical imaging, YOLO maintains high accuracy while processing images quickly, so it is particularly suitable for time-sensitive diagnostic tasks such as blood cell detection. For example, a recent YOLOv11-based model achieved 92.7% mAP (mean average precision) on the blood cell detection dataset (BCD), highlighting the high accuracy that modern YOLO architectures achieve in such applications [9,10].

YOLO has continuously evolved to the latest version (e.g., YOLOv11), which further enhances its applicability to complex tasks. YOLOv1 first introduced a real-time unified architecture for detection, and subsequent versions (v2–v4, v7, v8, v10, v11) gradually improved the processing capabilities for multi-scale objects under varying conditions [8,11]. As the latest iteration of the series, YOLOv11n adopts a lightweight yet high-performance design, to maximize computational efficiency, making it highly attractive for the microscopic detection process [12]. This makes YOLOv11n an ideal solution for blood cell detection, especially for problems such as cell overlap, lighting changes, and staining artifacts common in microscopic images.

Despite these advances, automated blood cell detection continues to face several challenges. Dense overlap of cells in microscopic fields of view can lead to missed or inaccurate detections, especially when cell boundaries are obscured by aggregation or smear artifacts [13]. Differences in sample preparation and staining across laboratories can lead to inconsistent image appearance, which affects the model’s ability to generalize to new clinical settings [14]. In addition, an imbalanced distribution of cell classes (e.g., RBCs are far more numerous than rare white blood cell subtypes) poses difficulties for training, as the model may be biased toward the dominant classes [15,16]. Many existing detection models suffer from high computational costs, limiting their deployment in urgent clinical settings where real-time or near-real-time analysis is required [17]. These limitations have motivated us to seek methodological improvements to enhance the accuracy, robustness, and efficiency of detection.

In this study, a method was proposed to address the aforementioned challenges by integrating gradient accumulation and loss reweighting into the YOLOv11n training pipeline. Gradient accumulation (with a linearly scaled learning rate) allows training with larger effective batch sizes using limited GPU memory, which may improve model convergence on complex data [18,19]. Task-specific loss reweighting is applied to balance the learning focus across object types and localization errors [20,21]. We hypothesize that these improvements will enhance YOLOv11n’s ability to detect blood cells in complex scenes (such as cell-dense areas and diverse imaging conditions) without changing the model’s inference architecture. The main goal is to demonstrate that YOLOv11n’s performance on blood cell detection can be improved by introducing gradient accumulation and loss reweighting. The main contributions of this study include:

- A training strategy is proposed that combines gradient accumulation with linear learning rate scaling and loss reweighting to improve the localization accuracy while keeping the YOLOv11n inference network unchanged.
- A reproducible evaluation system is established for the BCD dataset, using a fixed training/validation split and a standardized preprocessing.
- Compared with other compact detectors (YOLOv7-tiny, YOLOv8n, YOLOv9t, YOLOv10n), the improved YOLOv11n demonstrates a better accuracy-efficiency trade-off on the BCD dataset. This performance also outperforms that of prior models, including YOLOv10-l, CST-YOLO, and YOLOv7 using the BCCD dataset.
- Using ablation experiments and interpretability analysis (Eigen-CAM), it is demonstrated that gradient accumulation and loss reweighting provide complementary rather than simple additive effects.

The remainder of this paper is organized as follows: Section 2 describes the research methodology, including dataset characteristics, model architecture, and training optimization; Section 3 presents the experimental results, highlighting quantitative performance improvements and example detection results; Section 4 discusses the significance of the research findings, analyzes limitations, and suggests directions for future work; and, Section 5 concludes with a summary of the main contributions and their potential impact on clinical practice.

## 2. Materials and Methods

### 2.1. Blood Cell Detection Dataset

This study utilized the BCD dataset available on the Kaggle platform. The dataset contains annotated microscopic images with a resolution of 416 × 416 pixels. The images show the main classes of human blood cell samples, which are divided into three types: RBC, WBC, and platelet. Each image is accompanied by an annotation file listing the bounding box coordinates for each identified object and its class label (RBCs, WBCs, and platelets).

The BCD dataset is specifically designed to simulate the types of variations that may be encountered in real-world clinical diagnostic processes, and therefore the dataset is highly applicable to training models with strong robustness and generalization capabilities. This diversity is reflected in the differences in cell shape and size, which are due to differences between biological individuals and the different technical methods used in the preparation of blood smears. In addition, the dataset also takes into account the diversity of cell distribution, covering both the performance of sparse areas in blood smears and the characteristics of dense areas, thereby enhancing its applicability in actual diagnostic scenarios. In addition to the above factors, the dataset also contains images taken under different lighting conditions. These images show different staining intensities and contain artifacts or noise that are common in clinical microscopy. The robustness and generalization ability of the model can be further improved by intentionally introducing diverse imaging conditions, thereby enhancing its ability to cope with common challenges in the clinical environment.

**Figure 1:**
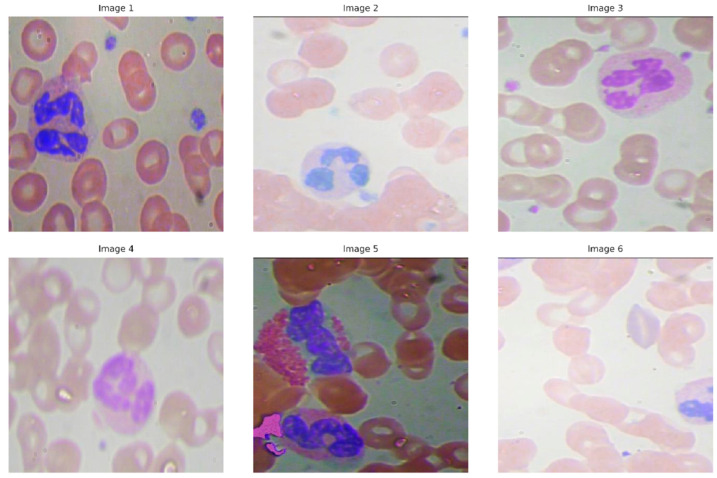
Randomly selected sample images from the BCD dataset.

The dataset is divided into two parts, a training set containing 765 images and a validation set containing 73 images. Figure 1 shows typical example images in the blood cell detection dataset, which intuitively reflects the diversity and complexity of the images used in the experiment.

### 2.2. Model Architecture of YOLOv11n

**Figure 2:**
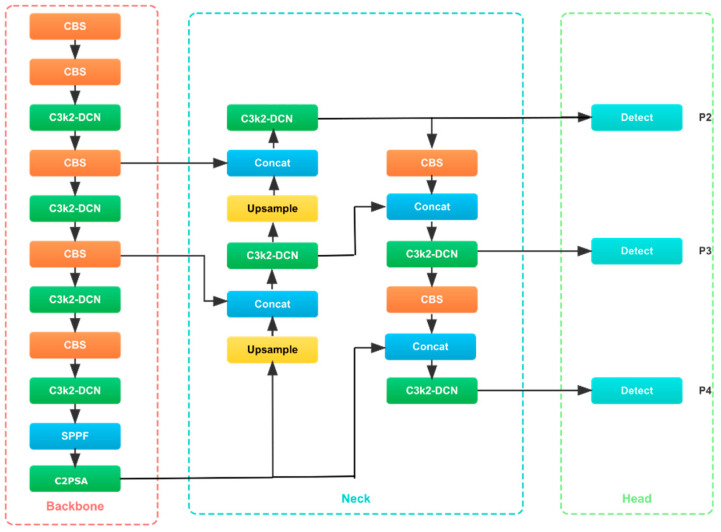
The structural diagram of the YOLOv11n model.

The YOLOv11n model architecture mainly consists of three components: the feature extraction module (Backbone), the feature fusion module (Neck), and the module that generates prediction results (Head). The architecture is optimized to meet the needs of high-performance object detection, so it has good adaptability in real-time applications, especially in the field of medical image analysis. The model adopts advanced technologies such as Cross Stage Partial with kernel size 2 (C3k2), fast spatial pyramid pooling (SPPF), cross-stage partial connection with spatial attention (C2PSA), and convolution-batch normalization-SiLU (CBS) module to effectively process multi-scale features and improve detection accuracy (see Figure 2).

The backbone first down samples the input image through a standard convolutional layer, which reduces the spatial dimension while increasing the number of feature channels. The operation of these layers can be expressed in mathematical form as:

(1) Y = X ∗ W + b

where X is the input image, W is the convolutional kernel, b is the bias, and Y is the output feature map. Following the convolution, the model incorporates C3k2 Blocks, which use two smaller convolutions instead of one large convolution, improving computational efficiency while maintaining performance. The operation inside a C3k2 block is defined as:

(2) $$C3k2(X)=\sigma \left(\left((X*W_{1})*W_{2}\right)+b\right)$$

where $W_{1}$ and $W_{2}$ are convolutional kernels, σ is the activation function, and b is the bias term. The C3k2 blocks enhance feature extraction while reducing computational costs.

SPPF (Spatial Pyramid Pooling Fast) follows, applying max-pooling at multiple scales to capture features from different spatial regions of the image. The pooling operation at each scale k is described as:

(3) $$P_{k}={\mathrm{MaxPool}}_{k}(X)=\max(X)\;\mathrm{for}\;k\in \{1,2,3\}$$

where $P_{k}$ is the pooled feature map at scale k. This multi-scale pooling allows the model to detect objects at various sizes.

C2PSA (Cross Stage Partial with Spatial Attention) is another critical module, enabling the model to focus on important regions in the image by applying spatial attention. This operation can be written as:

(4) A(X)=softmax(X)⊙X

where A(X) is the spatial attention map, and X is the feature map. By emphasizing key areas, the model improves its ability to detect small or occluded objects.

The Neck component integrates and refines features from multiple scales. It processes low, medium, and high-level features extracted by the Backbone and then upsamples and concatenates them. This process is mathematically represented as:

(5) $$P_{i+1}=\mathrm{Upsample}(P_{i})$$

where $P_{i}$ is the feature map at scale ii, and $P_{i+1}$ is the upsampled feature map at scale i+1. This allows the model to combine both high and low-level features for more accurate detection.

The C3k2 blocks in the Neck further refine these features, ensuring computational efficiency and robust feature fusion. The operation is similar to the one described for the Backbone:

(6) $$C3k2(X)=\sigma \left(\left((X*W_{1})*W_{2}\right)+b\right)$$

where X is the feature map to be processed. The attention mechanism in C2PSA further refines low-level features by focusing on spatially significant regions, expressed as:

(7) $$X_{\mathrm{att}}=A(X)⊙X$$

where $X_{\mathrm{att}}$ is the attention-modulated feature map, and A(X) is the spatial attention map.

The Head component generates final predictions by processing the features with C3k2 blocks. This final processing step can be written as:

(8) $${C3k2}_{\mathrm{final}}(X)=\sigma \left(\left((X*W_{1})*W_{2}\right)+b\right)$$

Afterward, CBS blocks (Convolution-BatchNorm-SiLU) are applied to stabilize the data flow, improve feature extraction, and apply the SiLU activation function. The CBS operation is:

(9) $$\mathrm{CBS}(X)=\sigma \left(\mathrm{BatchNorm}(X*W)\right)$$

where X is the input, W is the convolutional kernel, and σ is the SiLU activation function. Finally, the model predicts the bounding box coordinates and class labels through Conv2D layers:

(10) $$\hat{y}=\mathrm{Conv}2D(X)$$

where $\hat{y}$ represents the predicted output.

YOLOv11n’s architecture integrates efficient feature extraction, multi-scale fusion, and enhanced prediction generation. The mathematical operations in each component enable the model to manage intricate picture features, facilitating high-performance real-time detection applicable to several fields, including medical image analysis.

### 2.3. Strategies and Techniques for Optimization

This research enhances YOLOv11n training for blood cell detection with the integration of gradient accumulation and loss reweighting. The objective is to stabilize updates within memory constraints and to prioritize the training signal for accurate localization, while maintaining the inference-time architecture as described in Section 2.2.

Initially, gradient accumulation is employed to simulate a bigger effective batch size without augmenting memory usage [22]:

(11) $$g_{t}=\frac{1}{b}\sum_{i=1}^{b}\nabla_{w}l\;\left(x_{t,i},y_{t,i};w\right)$$

where $g_{t}$ is the gradient estimate at micro-step t; b is the micro-batch size; $x_{t,i}$ and $y_{t,i}$ are the i−th image and labels in micro-batch t; l(⋅;w) is the per-sample detection loss at parameters w; and $\nabla_{w}$ denotes the gradient with respect to w.

The accumulated gradient is then applied once every K micro-steps:

(12) $$w\leftarrow O\;\left(w,\frac{1}{K}G,\;\right),\;B_{\mathrm{eff}}=bK,\;G=\sum_{j=1}^{K}g_{t+j}$$

where O is the optimizer update rule (here, SGD with momentum); G is the accumulated gradient over K micro-steps; η is the learning rate; and $B_{\mathrm{eff}}$ is the effective batch size. In this work, b=16 and K=4, giving $B_{\mathrm{eff}}=64$.

The learning rate follows a linear-scaling rule to match the effective batch:

(13) $$\eta =\eta_{\mathrm{ref}}\cdot \frac{B_{\mathrm{eff}}}{B_{\mathrm{ref}}}$$

where $\eta_{\mathrm{ref}}$ is the base learning rate defined at reference batch $B_{\mathrm{ref}}$, and η is the scaled rate used at $B_{\mathrm{eff}}$.

Next, loss reweighting increases emphasis on localization while tempering penalties on visually similar classes [23]:

(14) $$L=\lambda_{\mathrm{box}}L_{\mathrm{box}}+\lambda_{\mathrm{cls}}L_{\mathrm{cls}}+\lambda_{\mathrm{dfl}}L_{\mathrm{dfl}}$$

where $L_{\mathrm{box}}$ is the bounding-box regression loss; $L_{\mathrm{cls}}$ is the classification loss; $L_{\mathrm{dfl}}$ is the distribution focal loss (DFL) used in the Head; and $\lambda_{\mathrm{box}},\lambda_{\mathrm{cls}},\lambda_{\mathrm{dfl}}$ are scalar weights. The weights are set to $\lambda_{\mathrm{box}}=9.0$, $\lambda_{\mathrm{cls}}=0.35$, and $\lambda_{\mathrm{dfl}}=1.0$, tuned for the accumulated setting.

Finally, both levers are realized in a single loop without altering the Backbone–Neck–Head design. Training runs for 120 epochs at image size 416×416 using SGD (momentum 0.937, weight decay 0.0005, with lr0=0.02 and final multiplier lrf=0.01. A 3-epoch warm-up raises momentum from 0.8 to 0.937 and warms the bias learning rate to 0.1. With b=16 and K=4, the configuration satisfies Equation (13) and produced +1.45% improvement over the baseline; Section 3 reports the full comparison.

### 2.4. Experimental Setup

#### 2.4.1. Environment and Training Parameters

Experiments were run on an HP Z8 G4 workstation (Intel Xeon Gold 6133, 128 GB DDR4 RDIMM ECC, NVIDIA Tesla V100) under Ubuntu 24.04 LTS with Python 3.12 and Ultralytics v8.3.179. A single GPU was used with a fixed random seed. The training parameters of YOLOv11n Optimized (YOLOv11n-Opt) are detailed in Table 1.

#### 2.4.2. Evaluation Protocols and Implementation

Evaluation used a fixed train/validation split (as defined in Section 2.1), identical image size (416×416), and identical preprocessing for all models. The checkpoint with the best validation mAP50 was selected and then reported on the held-out test set. Non-maximum suppression used the Ultralytics defaults. Inference measurements were taken on the Tesla V100, batch size 16, with confidence/NMS thresholds (conf = 0.25, iou = 0.7).

The primary and secondary metrics, along with complexity and speed, are defined below:

(15) $${\mathrm{mAP}}_{50}\;=\;\frac{1}{C}\sum_{c=1}^{C}{\mathrm{AP}}_{c}(\tau =0.50),\;{\mathrm{AP}}_{c}(\tau )\;=\;\int_{0}^{1}P_{c}(R;\tau )dR$$

where C is the number of classes; ${\mathrm{AP}}_{c}(\tau )$ is the area under the precision–recall curve $P_{c}(R;\tau )$ for class cc at IoU threshold τ; R is recall from 0 to 1.

(16) $${\mathrm{mAP}}_{50-95}\;=\;\frac{1}{|T|C}\sum_{\tau \in T}\sum_{c=1}^{C}{\mathrm{AP}}_{c}(\tau ),\;T=\{0.50,0.55,\ldots ,0.95\}$$

where T is the set of IoU thresholds from 0.50 to 0.95 in steps of 0.05; other symbols are as defined above.

(17) $$\mathrm{Parameters}\;(M)\;=\;\frac{\left|\Theta \right|}{10^{6}}$$

where |Θ| is the total count of learnable parameters in the network; values are reported in millions.

(18) $$\mathrm{GFLOPs}\;=\;\frac{1}{10^{9}}\sum_{l}{\mathrm{FLOPs}}_{l}$$

where ${\mathrm{FLOPs}}_{l}$ is the floating-point operation count of layer l for a single forward pass at image size 416 × 416; multiply–add is counted as two FLOPs.

(19) $$\mathrm{FPS}\;=\;\frac{N}{T_{\mathrm{total}}}\;=\;\frac{1}{\bar{t}}$$

where N is the number of test images processed, $T_{\mathrm{total}}$ is total wall-clock inference time, and $\bar{t}$ is average per-image latency; measurements use batch size 1 on the Tesla V100.

Implementation matched gradient accumulation (optimizer updates once per accumulation window; gradients zeroed on boundaries) and the learning-rate schedule that decays from lr0 to lrf·lr0. Per-epoch logs captured loss components (box, cls, dfl) and all metrics to support the analyses.

## 3. Results

### 3.1. Efficacy of YOLOv11n with Augmented Techniques

**Figure 3:**
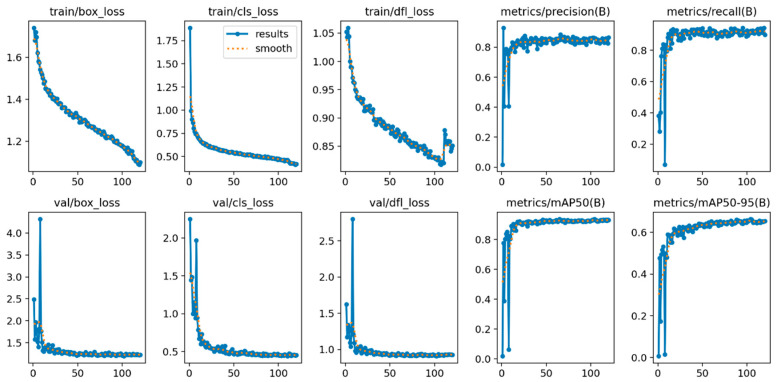
Training Loss and Metric Curves: Loss and performance metrics over training epochs, showing improvement in detection accuracy (The *x*-axis represents the training epochs, and the *y*-axis represents the corresponding loss or metric values).

**Figure 4:**
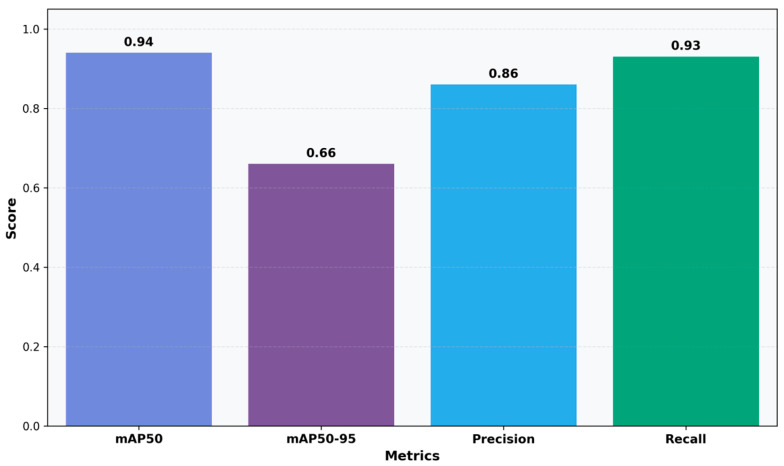
Detection Performance Metrics: Bar chart showing mAP50, mAP50-95, precision, and recall scores.

The training of YOLOv11n using advanced approaches shows consistent improvements in performance metrics and reductions in loss. The training loss curves (Figure 3) demonstrate a consistent decline in the box, classification, and dynamic focal loss metrics with time, signifying effective learning. Both precision and recall metrics (Figure 4) exhibit a similar trend, rapidly enhancing throughout training and stabilizing as the model nears convergence. The results indicate that the improved methods used for YOLOv11n model effectively facilitate the optimization process, enhancing its capacity to detect blood cells.

**Figure 5:**
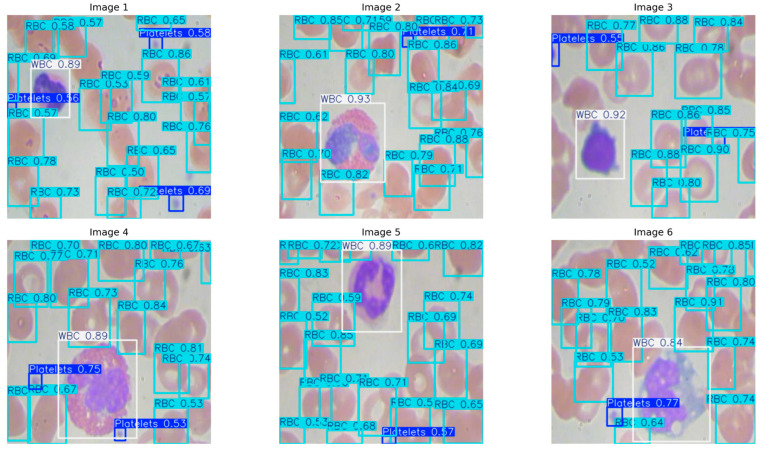
Qualitative detection on six validation images.

The qualitative results from six preserved smear patches (Figure 5) correspond to the measurements. The model precisely localizes individual WBCs with high confidence (≈0.84–0.93) and clear borders, tracks densely clustered RBCs despite considerable overlap with variable confidence scores (≈0.50–0.91), and reliably identifies small platelets with consistent scores (≈0.53–0.77). Detections persevere with image edges and under partial occlusion, with sporadic low-confidence bounding boxes in densely packed regions of RBCs. In evaluating the model’s quantitative detection efficacy, YOLOv11n-Opt exhibits strong performance, achieving an mAP50 of 0.9356, an mAP50–95 of 0.6620, a precision of 0.8604, and a recall of 0.9251. These numbers illustrate the model’s robust ability to precise blood cell detection. The mAP50 indicates strong effectiveness in identifying blood cells in ideal circumstances, while the mAP50-95 score underscores the model’s adaptability in more challenging detection scenarios. The balance between precision and recall stresses the model’s efficacy in reducing both false positives and false negatives, rendering it potentially suitable for clinical applications where accuracy is critical.

**Figure 6:**
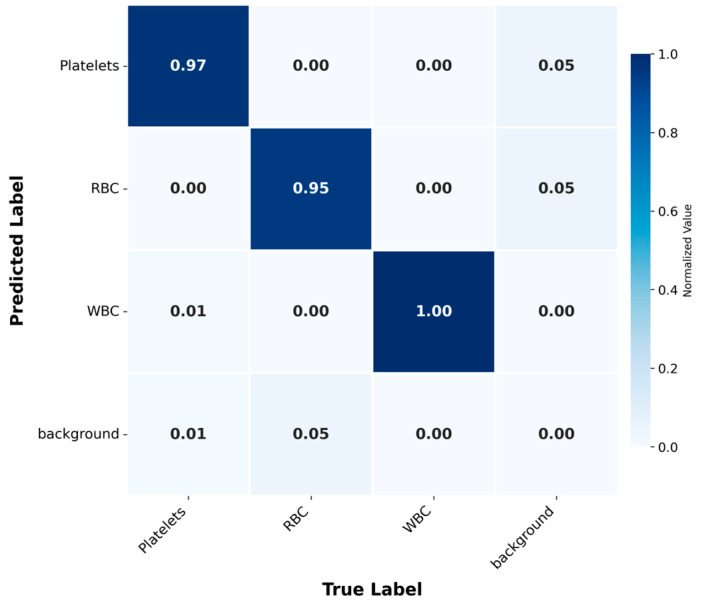
Confusion Matrix: Normalized matrix displaying the classification accuracy for blood cell types and background.

Further analysis of the confusion matrix (Figure 6) confirmed the high performance of the model, with classification accuracies of 0.97, 0.95 and 1.00 for platelets, RBCs and WBCs, respectively. The background category was identified with high accuracy, and misclassification was rare. This result indicates that the model has the ability to effectively distinguish between blood cell types and background. This high-precision classification is of great significance for clinical diagnosis, because the accurate distinction between these categories may directly affect the treatment effect of patients.

The YOLOv11n-Opt model demonstrates high efficacy in blood cell detection. Improved methods make training more consistent and the results more accurate. This model represents a viable method for analyzing medical images due to its superior accuracy, precision, and robust performance. It may also aid in detection blood cells in clinical settings.

### 3.2. Comparative Performance Analysis

This section compares YOLOv11n-Opt, using gradient accumulation and loss reweighting strategies, with other YOLO models: YOLOv7-tiny, YOLOv8n, YOLOv9t, and YOLOv10n. The assessment used the BCD dataset and focused on performance metrics including mAP50, mAP50–95, model parameters, GFLOPs, and FPS.

The findings indicate that YOLOv11n-Opt outperforms other models across most measures (Table 2). Its mAP50 reached a peak value of 0.9356, surpassing YOLOv8n (0.9179), YOLOv9t (0.9217), and YOLOv10n (0.8873), indicating higher detection accuracy. Moreover, YOLOv11n-Opt exhibits high computational efficiency, requiring 6.6 GFLOPs and achieving 106.9 FPS, indicating improved inference performance relative to the other models.

Moreover, class-specific performance assessments indicate that YOLOv11n-Opt demonstrates superior capability in identifying distinct blood cell types (Table 3). The model attains the maximum WBC_AP50 of 0.9311, RBC_AP50 of 0.8894, and Platelets_AP50 of 0.9864. These numbers further underscore the model’s efficacy in identifying blood cells, especially for the more complex WBC and RBC categories, in comparison to other models in the study. YOLOv11n-Opt uses the YOLO11n backbone (2.6 M parameters; 6.6 GFLOPs @640), enabling real-time GPU inference and modest CPU requirements.

In conclusion, YOLOv11n-Opt exhibits exceptional efficacy in blood cell detection, merging high precision with computational efficiency. The model attains superior detection scores in mAP and provides exceptional FPS, rendering it extremely appropriate for real-time clinical applications. Moreover, its superior detection capabilities, especially for WBC and RBC, establish it as a premier solution in blood cell analysis.

### 3.3. Ablation Study

**Figure 7:**
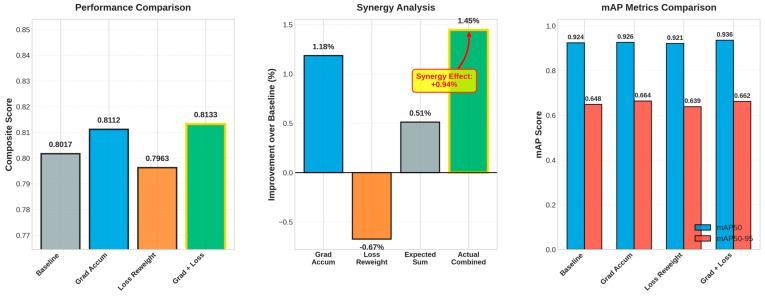
Performance comparison, improvement and synergy analysis, and mAP metrics bars.

**Figure 8:**
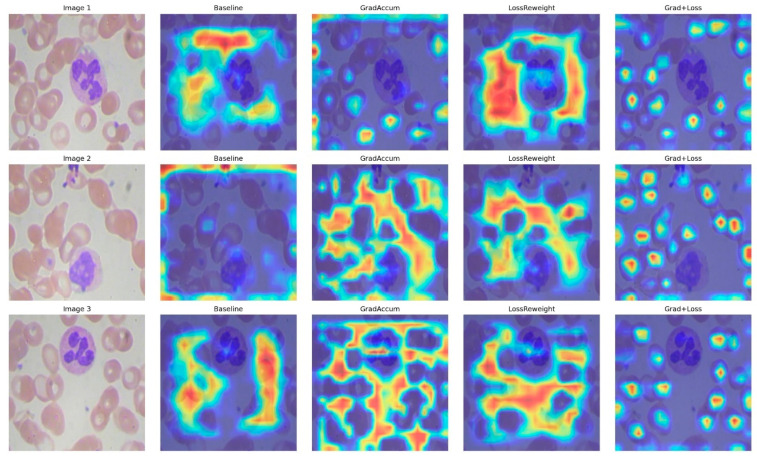
Eigen-CAM comparisons for Baseline, Grad Accum, Loss Reweight, and Grad + Loss across three representative images.

This section assesses four configurations of YOLOv11n on the blood-cell dataset: Baseline, Gradient Accumulation, Loss Reweighting, and the integrated approach (Grad + Loss). Quantitative results are shown in Table 4, while metric and interaction plots are illustrated in Figure 7, accompanied by qualitative Eigen-CAM evidence in Figure 8.

Initially, when evaluating each strategy independently, Gradient Accumulation enhanced overall detection quality compared to the baseline, elevating the composite score from 0.8017 to 0.8112 (+1.18%), with a significant improvement in mAP50-95 (0.6485 → 0.6641, +2.41%) and a notable increase in recall (0.9145 → 0.9451), albeit with a minor decline in precision (0.8525 → 0.8402). In contrast, Loss Reweighting alone diminished performance across all metrics (composite 0.7963, −0.67%; mAP50 0.9241 → 0.9210, −0.34%; mAP50-95 0.6485 → 0.6389, −1.48%), suggesting that reweighting without supplementary stabilization disproportionately highlighted challenging or infrequent cases, thereby disrupting the established equilibrium.

In the integrated setup, the Grad + Loss model achieved best results, achieving a composite score of 0.8133 (+1.45%), mAP50 of 0.9356 (+1.24%), and mAP50–95 of 0.6620 (+2.08%). It improved precision (0.8604) and recall (0.9251) compared with the baseline. The observed composite improvement (+1.45%) exceeded the anticipated additive gain (+0.51% = 1.18% − 0.67%), yielding a synergy of +0.94% (Figure 7). This suggests that gradient accumulation stabilizes the optimization, enabling loss reweighting to prioritize underrepresented or ambiguous cell instances without undermining calibration.

To understand why the integrated technique yields optimal results, Eigen-CAM visualizations reveal that the baseline exhibits dispersed attention and frequently encroaches on background erythrocytes (Figure 8); Gradient Accumulation alone results in wider yet more uniform activation, occasionally accompanied by peripheral artifacts; Loss Reweighting alone focuses on a limited number of high-contrast areas, neglecting boundaries; the integrated model generates compact, nucleus-centric attention while diminishing background responses and achieving clearer delineation in congested environments, and consistent with the enhanced precision, sustained recall, and elevated mAP50–95. The lack of focus on WBCs is due to the model detecting them using different, lower-level features that may not correspond to the high-activation regions shown in Eigen-CAM.

## 4. Discussion

The performance enhancements noted in this study result from two training mechanisms—gradient accumulation and loss reweighting—implemented without modifying the YOLOv11n Backbone–Neck–Head or its inference-time configurations. Gradient accumulation enhances the optimization under memory constraints by averaging micro-batch gradients before each update, thereby diminishing update noise and facilitating convergence in densely populated areas. Loss reweighting amplifies the impact of localization and DFL relative to classification, refining bounding boxes while maintaining class calibration in visually analogous cell instances. When used together, these mechanisms enhance mAP and the precision-recall balance compared with the baseline and are consistent with the training curves, confusion matrix, and ablation findings presented in Section 3. The architecture and forward pass remain intact, meaning that inference-time computation and deployment costs remain unchanged. The conclusions are constrained by the scale of the dataset and potential domain shift; external validation using additional data is essential.

In a comparison of the YOLOv11n-Opt model with established methods (Table 5), including those by several researchers, the YOLOv11n-Opt consistently outperforms them on key metrics [9,24]. One model attains higher classification accuracy for RBCs, platelets, and WBCs; however, YOLOv11n-Opt performs better, with greater mAP50, precision, and recall. That model’s classification accuracies for RBCs, platelets, and WBCs are 0.874, 0.874, and 0.998, respectively, whereas YOLOv11n-Opt attains an mAP50 of 0.936 across these categories. The CST-YOLO, reported to have an overall score of 0.911, falls short of that of the YOLOv11n-Opt model. In a comparison with other models applied to the Blood Cell Count and Detection (BCCD) dataset, YOLOv7 achieved an overall mean mAP of 0.896, while the recently introduced YOLOv10-l model achieved a higher mAP of 0.916, although it remains less efficient. These findings further highlight the superior performance of the YOLOv11n-Opt model. These comparative results highlight the effectiveness of the YOLOv11n in clinical blood cell detection, especially in complex real-world scenarios. The superior performance of YOLOv11n-Opt, demonstrated through higher mAP50, precision, and recall compared to established models, positions it as a more reliable and efficient tool for automated blood cell analysis. This enhanced accuracy can significantly streamline diagnostic workflows in medical laboratories, leading to faster and more precise identification of blood cell types in clinical settings. Although the results are encouraging, this study still has some limitations, including a small size of the dataset and potential difficulty generalizing to different clinical environments. In addition, fluctuations in imaging quality or the presence of artifacts may affect model performance. Moreover, the computational expense of training, especially with gradient accumulation, limits the model’s availability in resource-constrained settings.

The model applicability maybe limited when blood smears differ from the training domain (e.g., stains, magnifications, scanners), where domain shift typically reduces accuracy. It also degrades on very small or overlapping targets (e.g., platelets) and in low-resolution or artifact-laden fields, reflecting persistent small-object and occlusion limits. Subsequent research should focus on augmenting the dataset to encompass a broader range of blood cell types and varied imaging settings. Exploring alternative augmentation methods and refining the model for faster inference may enhance its clinical utility. The model cannot detect some small cells (platelets), and this should be an area of attention in future work. Building a real-time system and integrating the optimized YOLOv11n model into a fully automated diagnostic instrument can significantly improve clinical diagnostics and help identify blood cell abnormalities more quickly and accurately, thereby improving patient care.

## 5. Conclusions

In this study, an enhanced blood cell detection model named YOLOv11n-Opt is proposed to improve the accuracy and robustness of blood cell detection for clinical diagnosis. By introducing gradient accumulation and loss reweighting techniques, the performance of the YOLOv11n-Opt on the blood cell detection dataset has been significantly improved, specifically in terms of mAP50, mAP50-95, precision, and recall. Detailed ablation experiments verified the respective contributions of these strategies. The study shows that the integration of the two techniques has a significant effect on improving detection accuracy, especially for challenging cell types such as WBCs and platelets. In addition, the model demonstrates strong adaptability to various clinical imaging conditions, including cell overlap, occlusion, and different lighting conditions. The main findings are as follows:

1. The gradient accumulation and loss reweighting method can significantly enhance the stability of the training process and effectively improve detection performance, especially when dealing with challenging cases with a small number of blood cells, and the method does not significantly affect computational efficiency.
2. The performance of YOLOv11n-Opt exceeds that of current methodologies, including the recently proposed updates. The model shows higher values in mAP50 and mAP50-95 for all blood cell types, and shows better performance in precision and recall.
3. The model can effectively cope with diverse clinical imaging conditions, such as different cell distributions and cell overlap, and is therefore regarded as a reliable tool for blood cell detection in clinical scenarios.
4. Through systematic ablation experiments, the study shows that there is complementarity between gradient accumulation and loss reweighting. The synergy between the two can further improve the model robustness without reducing the model accuracy.

The results of the YOLOv11n-Opt show that the integration of advanced techniques such as gradient accumulation and loss reweighting into a mature object detection architecture can significantly improve the accuracy and efficiency of medical image analysis. This study lays the foundation for the development of a more reliable and real-time blood cell detection system, which will help identify blood cell abnormalities in a timely and accurate manner for clinical diagnosis, thereby improving patient prognosis.

## Figures and Tables

**Table 1:** Training parameters (YOLOv11n-Opt).

Model	Epochs	Image Size	Micro-Batch/Accum (Eff. Batch)	Optimizer	lr0/lrf	Momentum/Weight Decay	Loss Weights (box/cls/dfl)
YOLOv11n	120	416 × 416	16/4 (64)	SGD	0.02/0.01	0.937/0.0005	9.0/0.35/1.0

**Table 2:** Performance comparison of YOLOv11n-Opt with four models across key metrics, including mAP, model parameters, GFLOPs, and FPS.

Model	mAP50	mAP50–95	Parameters (M)	GFLOPs	FPS
YOLOv8n	0.9179	0.6417	3.16	8.9	37.2
YOLOv9t	0.9217	0.6456	2.13	8.5	38.0
YOLOv10n	0.8873	0.6346	2.78	8.7	65.8
YOLOv11n-Opt	0.9356	0.6620	2.62	6.6	106.9

**Table 3:** Class-specific AP50 comparison for WBC, RBC, and Platelets detection across YOLO models.

Model	WBC_AP50	RBC_AP50	Platelets_AP50
YOLOv8n	0.9033	0.8710	0.9793
YOLOv9t	0.9038	0.8763	0.9851
YOLOv10n	0.8381	0.8553	0.9685
YOLOv11n-Opt	0.9311	0.8894	0.9864

**Table 4:** Ablation summary (validation set).

Configuration	mAP50	mAP50–95	Precision	Recall	Composite
Baseline	0.9241	0.6485	0.8525	0.9145	0.8017
Grad Accum	0.9260	0.6641	0.8402	0.9451	0.8112
Loss Reweight	0.9210	0.6389	0.8516	0.9115	0.7963
Grad + Loss	0.9356	0.6620	0.8604	0.9251	0.8133

**Table 5:** Model performance comparison on blood cell detection (mAP50).

Model	RBCs	WBCs	Platelets	Overall
YOLOv10n [9]	0.874	0.998	0.874	0.912
CST-YOLO [24]	0.857	0.899	0.978	0.911
YOLOv7 [25]	0.829	0.977	0.883	0.896
YOLOv10-l [10]	0.927	0.882	0.984	0.916
YOLOv11n-Opt	0.931	0.889	0.986	0.936

## Data Availability

The training data consisted of images sourced from the Blood Cell Detection Dataset (BCD) published on the Kaggle platform (https://www.kaggle.com/datasets/adhoppin/blood-cell-detection-datatset, accessed on 25 September 2025).
